# Understanding determinants of vaccine hesitancy and acceptance in India: A qualitative study of government officials and civil society stakeholders

**DOI:** 10.1371/journal.pone.0269606

**Published:** 2022-06-09

**Authors:** Daniel J. Erchick, Madhu Gupta, Madeleine Blunt, Adarsh Bansal, Molly Sauer, Amelia Gerste, Taylor A. Holroyd, Brian Wahl, Mathuram Santosham, Rupali J. Limaye

**Affiliations:** 1 Department of International Health, Johns Hopkins Bloomberg School of Public Health, Baltimore, Maryland, United States of America; 2 Department of Community Medicine and School of Public Health, Postgraduate Institute of Medical Education and Research, Chandigarh, India; 3 International Vaccine Access Center, Johns Hopkins Bloomberg School of Public Health, Baltimore, Maryland, United States of America; 4 Department of Epidemiology, Johns Hopkins Bloomberg School of Public Health, Baltimore, Maryland, United States of America; 5 Department of Population, Family and Reproductive Health, Johns Hopkins Bloomberg School of Public Health, Baltimore, Maryland, United States of America; 6 Department of Pediatrics, Johns Hopkins School of Medicine, Baltimore, Maryland, United States of America; 7 Department of Health, Behavior & Society, Johns Hopkins Bloomberg School of Public Health, Baltimore, Maryland, United States of America; ICMR-National Institute for Research in Tuberculosis: National Institute of Research in Tuberculosis, INDIA

## Abstract

**Introduction:**

Few studies have described the drivers of vaccine hesitancy and acceptance in India from the perspective of those involved in the design and implementation of vaccine campaigns–such as government officials and civil society stakeholders–a prerequisite to developing approaches to address this barrier to high immunization coverage and further child health improvements.

**Methods:**

We conducted a qualitative study to understand government officials and civil society stakeholders’ perceptions of the drivers of vaccine hesitancy in India. We conducted in-depth phone interviews using a structured guide of open-ended questions with 21 participants from international and national non-governmental organizations, professional associations, and universities, and state and national government–six national-level stakeholders in New Delhi, six state-level stakeholders in Uttar Pradesh, six in Kerala, and three in Gujarat–from July 2020 to October 2020. We analyzed data through a multi-stage process following Grounded Theory. We present findings on individual-level, contextual, and vaccine/vaccination program-specific factors influencing vaccine hesitancy.

**Results:**

We identified multiple drivers and complex ways they influence vaccine beliefs, attitudes, and behaviors from the perspective of government officials and civil society stakeholders involved in vaccine campaigns. Important individual-level influences were low awareness of the benefits of vaccination, safety concerns, especially related to mild adverse events following immunization, and mistrust in government and health service quality. Contextual-level factors included communications, the media environment, and social media, which serves as a major conduit of misinformation and driver of hesitancy, as well as sociodemographic factors–specific drivers varied widely by income, education, urban/rural setting, and across religious and cultural groups. Among vaccine/vaccination-level issues, vaccine program design and delivery and the role of health care professionals emerged as the strongest determinants of hesitancy.

**Conclusions:**

Drivers of vaccine hesitancy in India, as elsewhere, vary widely by local context; successful interventions should address individual, contextual, and vaccine-specific factors. While previous studies focused on individual-level factors, our study demonstrates the equal importance of contextual and vaccine-specific influences, especially the communication and media environment, influential leaders, sociodemographic factors, and frontline health workers.

## Introduction

Vaccination is one of public health’s most critical tools, responsible for preventing 2–3 million deaths every year, with potential to prevent an additional 1.5 million deaths through increased coverage [1]. Yet vaccine hesitancy–the reluctance or refusal to vaccinate despite availability of vaccines–poses an urgent threat to vaccine success [2]. Globally, vaccine hesitancy is complex, context specific, and driven by multiple influences [3, 4]. Several conceptual models have been proposed to understand the drivers of vaccine hesitancy, including the 5Cs model (confidence, complacency, constraints, calculation, and collective responsibility) and the more complex Matrix of Hesitancy Determinants from the Strategic Advisory Group of Experts on Immunization (SAGE) [2, 5]. Common reasons for vaccine hesitancy are safety concerns, including side effects; lack of knowledge and awareness of the importance of vaccination; and religious, cultural, gender, and socioeconomic issues [4]. Vaccine hesitancy, and conversely, acceptance or confidence, are individual phenomenon as well as social and political phenomenon, increasingly influenced by social media, public figures, and popular movements [6]. Despite the importance of this issue, few countries, particularly in low resource settings, have rigorously characterized the determinants of hesitancy or developed effective approaches to monitor and address vaccine hesitancy.

In recent years, India’s national immunization program–the Universal Immunization Programme (UIP), the largest in the world, with an annual birth cohort of 27 million children [7]–has achieved several vaccination-related benchmarks. These include polio-free certification in 2014 and elimination of maternal and neonatal tetanus transmission in 2015. India launched a nationwide effort, Mission Indradhanush, in 2014 to increase vaccination in hard-to-reach areas, and has introduced a second dose of measles-containing vaccine and five other vaccines into the UIP since 2010 (Hib-containing pentavalent vaccine, inactivated polio vaccine (IPV), rotavirus vaccine (RVV), measles-rubella (MR) vaccine, and pneumococcal conjugate vaccine (PCV)) [8]. Despite these achievements, only 62% of children 12–23 months were fully immunized (three doses of diphtheria-tetanus-pertussis (DTP) vaccine, three doses of polio vaccine, one dose of Bacillus Calmette–Guérin (BCG) vaccine, and one dose of measles vaccine) according to the National Family Health Survey (NFHS) 2015–16; further, coverage ranged widely by state/union territory, from 35% in Nagaland to 91% in Puducherry, with substantial inequities by geography, socioeconomic factors, and access to care [9, 10].

As India has worked to increase immunization coverage through supply-side approaches (e.g., human resources, supply logistics, cold chain, etc.), demand-side challenges, especially vaccine hesitancy, have increasingly become a priority for health officials, civil society partners, and media. The country has experienced several high-profile challenges related to vaccine hesitancy; most notably, in 2017–2019, during India’s MR vaccine introduction campaign, vaccine refusal arose rapidly in several communities, driven by inadequate pre-campaign communications planning and widespread misinformation and rumors on social media [11–13]. However, little has been documented regarding the determinants of vaccine hesitancy in these communities, the series of events that led to these incidents, or lessons learned about how to prevent or manage such occurrences in the future [14].

Understanding the drivers of hesitancy generally, and lessons learned from high-profile campaigns such as India’s MR introduction, is critical to developing evidence-based, country-led strategies to increase vaccine acceptance and reduce the burden of vaccine-preventable disease [15]. The few studies that have sought to describe the determinants of vaccine hesitancy in India varied widely in location, focus, and time frame [14]. These studies have focused on attitudes of the target audience. To our knowledge, little research has been conducted that examines the perceptions of those involved in the design and implementation of vaccine campaigns. Our aim for this qualitative study was to broadly characterize the drivers of vaccine hesitancy and acceptance and identify potential interventions to address these challenges, drawing on perceptions of government health officials and civil society stakeholders from the national level and three states (Uttar Pradesh, Kerala, and Gujarat).

## Methods

### Study design

We conducted a qualitative study of in-depth interviews following Grounded Theory from July 2020 to October 2020 to understand government officials and civil society stakeholders’ perceptions of vaccine knowledge, attitudes, and drivers of vaccine hesitancy. We used purposive sampling to identify professionals with experience in implementation of routine vaccination programs and mass campaigns from the national level and in three states in India. We selected three states–Uttar Pradesh, Kerala, and Gujarat–to represent distinct regions with diverse experiences related to the MR vaccination campaign and unique challenges related to vaccine hesitancy. Interviewees represented a range of professions and diverse expertise, including pediatricians, program managers, communications experts, academics, and current or former government officials, with knowledge of India’s MR introduction campaign or vaccine acceptance and hesitancy in the country generally. We continued recruitment until we achieved data saturation related to our primary research question of broadly describing the drivers of vaccine hesitancy and acceptance and potential interventions to address these challenges in India.

### Data collection

Interviews followed a semi-structured interview guide. Topics included the extent and nature of vaccine hesitancy, relationships between sociodemographic factors and vaccine attitudes and beliefs, and how planning and response of government and partners and factors like anti-vaccine voices and social media impact vaccine hesitancy and acceptance. Interviews were conducted in English via Zoom audio and lasted 45 to 75 minutes. Discussions were recorded and audio files were transcribed using an online software tool. Transcripts were compared to the original audio files by two team members to correct any mistakes in transcription made by the software. Oral consent was obtained from all participants. This study received ethical approval from the Institutional Review Board at the Johns Hopkins Bloomberg School of Public Health (Baltimore, USA) and the Institutional Ethics Committee at the Postgraduate Institute of Medical Education and Research (Chandigarh, India).

### Data analysis

We analyzed data thematically through a multi-stage process following the constant comparative method in Grounded Theory [16]. This iterative process began with an open coding exercise of a single random interview by five team members. Open coding was followed by group discussion to develop an initial code structure. The team used this code structure to code a second transcript and revise the code structure. A smaller group of three team members coded a third transcript and finalized the code list. The final code list is based upon a socio-ecological model, including three major domains adapted from the SAGE Matrix of Hesitancy Determinants–contextual factors, individual and group factors, and vaccine or vaccine-specific issues–each with five subdomains. The code list was then applied to all transcripts by two team members for the next stage of analysis. Themes that emerged from this process were reviewed and categorized by code. The larger team met to discuss the main results and agree to their interpretations and conclusions. Data were analyzed in ATLAS.ti 9 (Scientific Software Development GmbH, Berlin, Germany).

## Results

**Fig 1:**
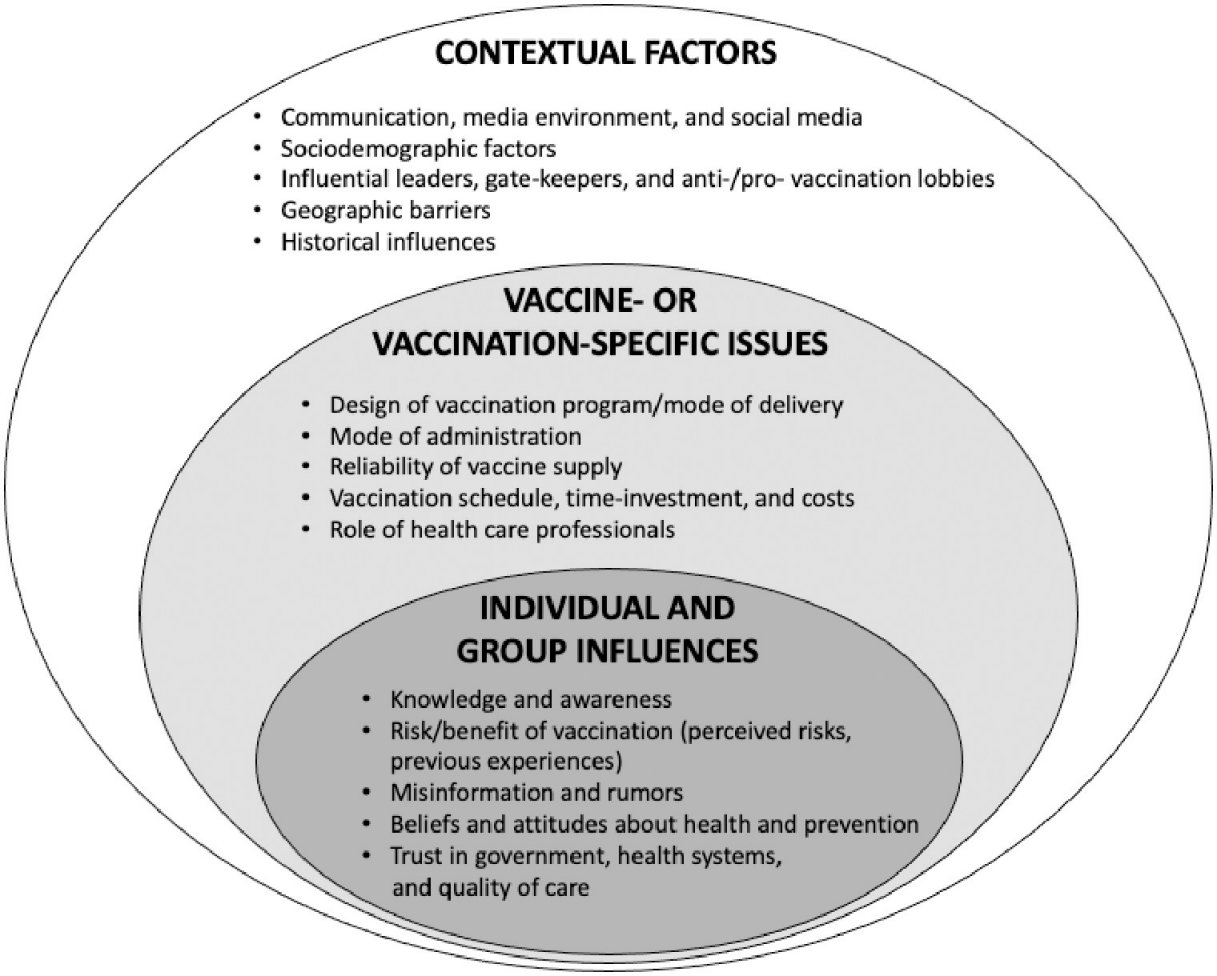
A matrix of the determinants of vaccine hesitancy in India adapted from the Matrix of Hesitancy Determinants by the Strategic Advisory Group of Experts on immunization (SAGE).

We contacted 40 individuals and 21 agreed to participate, consented, and were interviewed, including government officials and civil society stakeholders from international and national non-governmental organizations, professional associations, and universities. The sample included five (24%) women and 16 men (76%) with six national-level stakeholders in New Delhi, six state-level stakeholders in Uttar Pradesh, six in Kerala, and three in Gujarat. Their perceptions of the drivers of vaccine hesitancy in India fit with the broad categories defined by the SAGE matrix–individual and groups influences, contextual influences, and vaccine/vaccination-specific issues (Fig 1). Individual and group influences concern personal and social/peer factors, such as knowledge, beliefs, and attitudes, including those shaped by previous experiences with vaccination; contextual influences are those that arise from sociodemographic, culture, historical, or systematic factors; and vaccine/vaccination-specific issues relate directly to factors like delivery, access, and administration.

### A. Individual and group influences

#### Knowledge and awareness

Understanding of vaccines and the diseases they protect against is limited in most communities. There is a general understanding of vaccination with little knowledge about specific vaccines. This “knowledge gap” was identified as a leading determinant of vaccine hesitancy across all three states.

*Many people are fully aware of the importance of vaccination for their children and not many people seek vaccination on their own*

*(Key informant interview (KII)-11)*.

*The root of all these problems is lack of information and the remedy for this is also providing the information in a correct way*

*(KII-1)*.

However, there is a basic awareness among parents of certain vaccines because of their mode of administration (e.g., OPV), common adverse events following immunization (AEFIs) (e.g., DTP), or delivery in recent introduction or mass campaigns (e.g., OPV, MR).

*There’s definitely a messaging in the community that there are vaccines [like DTP that] cause fever*. *So*, *people would say*, “*Please don’t give that fever vaccine*”

*(KII-19)*.

Awareness of vaccines to prevent some causes of pneumonia and diarrhea was low prior to the recent introductions of the rotavirus and pneumococcal conjugate vaccines into the UIP.

*Until quite recently*, *[even professionals in the health sector] were not aware that diarrhea can be prevented by vaccines*. *Similarly*, *they were not aware that the pneumonia can be prevented by vaccines*

*(KII-20)*.

Low incidence of vaccine preventable diseases was suggested as a reason for low awareness of the importance of vaccines and a contributor to hesitancy.

*People are asking for TT [vaccine]*, … *every injury they want the injection*. *Because they’re afraid of tetanus disease*. *And they’re also afraid of rabies disease*. … *But other vaccines they reject*, *[like] measles*, *rubella [vaccine]*

*(KII-8)*.

### Risk/Benefit of vaccination (perceived risks and previous experiences)

Personal, family, or community experiences with vaccination, especially fear of common mild side effects, such as injection site redness or pain or low fever, were important drivers of vaccine hesitancy in all states.

*[Parents]* … *initially do not refute* … *vaccination*, *but later when the child gets sick*, *especially [from] fever [due to] pentavalent [vaccine] or* … *pain at the site of the injection*, *then they drop out*

*(KII-3)*.

Concerns related to AEFIs were also driven by the impact on parents’ ability to work or interpersonal issues.

“*I brought the child [to be] vaccinated*. *The child was crying*. *The child had fever*. *My husband came [home] drunk and was not able to sleep*. *So [my] husband beat me the entire night because the child was crying*. *So why should I now take the child to get vaccinated*?”

(*KII-6*)

Health professionals often do not sufficiently explain to parents about side effects to expect, how long they will last, and what people can do about them. Participants noted that paracetamol is not always provided, and increased coverage of this intervention could help reduce vaccine hesitancy.

*I tried to contact the health team*, *but nobody was available*. *I didn’t know where to go*. *So*, *I had to go to a private practitioner*, *and then it was*, *actually*, *it was simple*, *a slight swelling and the pain*, *redness*, *and pain was there*, *which was very much natural in a benevolent injection*.” … *Advice was not given well by the health team and [neither was] the painkiller*. … *So they went to a private practitioner* … *and they had to spend 500 rupees*

*(KII-1)*.

Witnessing a sick child after immunization was described as having an impact on vaccination attitudes beyond that individual child, extended to their siblings, neighbors, or even the wider community.

*If there is already a case [AEFI]*, *which has been* … *registered in the area of the child [who] got vaccination*, *and something happened to that child*, *that entire community would develop a kind of resistance*

*(KII-19)*.

There were also concerns about receiving multiple injections in one day.

*Not all mothers*, *but* … *definitely a percentage of them*, *would feel that the child should not get too many shots in one day*.

*(KII-19)*.

#### Misinformation and rumors

Participants shared examples of common misinformation and rumors about harmful effects of vaccines and vaccination. One rumor, which had circulated for years related to polio vaccine and resurfaced during the MR introduction campaign, was that vaccines were being administered to sterilize Muslim children to control growth of this population.

*[One rumor was that] this particular vaccine [MR] is designed to make the particular religion weak and harm their devotees* … *[or] make particular religion devotees sterile*

*(KII-12)*.

There were also examples of rumors circulating outside the context of the MR campaign. For example, in Kerala, there were rumors that tetanus toxoid vaccine could cause an abortion.

*Abortion occurs usually in the second month*, … *people attribute it*, *even [some] doctors attribute these abortions*, *to tetanus toxoid vaccine*

*(KII-8)*.

There were several conspiracy theories, especially during the MR campaign, about plots by foreign entities or the local government to harm specific communities.

*Some* … *believe* … *rumors*, *especially in the minority community* … *that government is doing this because they want to sterilize the community*

*(KII-17)*.

*They are picking up stories coming from the West* … *They say that it is* … *a particular foreign agent or foreign molecule*, *which is being introduced against our own immune system*

*(KII-20)*.

There were rumors that newer vaccines, such as the MR vaccine, are not really beneficial to the community and are being pushed for corporate profit.

*People think that these newer vaccines are only for the private sector for money making*, *and so there are now many conspiracy theories for many years blaming companies or philanthropic organizations*

(*KII-14*).

#### Beliefs and attitudes about health and prevention

Participants described relationships between vaccine hesitancy and non-allopathic medical belief systems, largely naturopathy in Kerala. Some followers of naturopathy believe that vaccination is unnecessary. Others claim that vaccination is unnatural, harmful, or “Western.”

*They said* … *if you follow naturopathy you don’t require any vaccination at all*

*(KII-13)*.

*They deny that this is due to the viruses or bacteria*, *and there is no need for any vaccination or preventive strategy*

*(KII-2)*.

#### Trust in government, health systems, and quality of health care services

Trust in government and perceptions of the quality of public health facilities and services contribute in multiple ways to vaccine hesitancy. A common perception is that health care services provided by government are of lower quality than private facilities.

*If government is not providing proper services to particular village if* … *water or sanitation* … *are not there*, *people will start avoiding the government programs*. *They don’t understand that this is important for them* … *and they will avoid* … *the immunization program*

*(KII-17)*.

*The highly educated and financially well to do* … *they think that the public health facility or the government facilities are only for the poor*

*(KII-3)*.

A perception in some communities that government highly prioritizes vaccination, while other health and infrastructure priorities are seemingly neglected, is a source of mistrust.

*[For] something [that] is delivered through the government system–for example*, *the polio vaccine or new MR vaccine campaign–to the community*, *[there is a perception] that [if] these vaccines are delivered to us at our doorstep free of cost*, *there may be some underlying agenda*

*(KII-20)*.

Previous experiences with government services also impact perceptions of vaccination.

*It’s just not vaccination*. *Maybe their past experiences have been bad with the [government] service delivery of any such kind*

*(KII-16)*.

### B. Contexual factors

#### Communication, media environment, and social media

Social media is a major conduit of misinformation, posing serious challenges in the recent MR introduction campaign. Misinformation, spread through social media, can trigger a “chain reaction” that rapidly changes vaccine attitudes and behaviors.

*You see in the MR campaign social media was full of negative messages*. … *WhatsApp messages* … *fuel this ongoing resistance campaign*

*(KII-20)*.

*Usually*, *people do not follow true messages*, *they tend to follow only messages which have some attraction*. *So all these negative messages get full support and they’re getting forwarded repeatedly*. *And whenever they say the vaccine contains something*, *which we should not have taken so it is against our religion*, *people tend to follow that message*

*(KII-13)*.

Misinformation is present in all types of media, including messages, infographics and memes, and videos. Videos are particularly impactful as they can be understood by all, including those who cannot read.

*On the very second day of the campaign* … *an older [video] clip from a news channel that was* … *forged [manipulated] [began circulating]*. *It was presented as if this happened during this campaign*. *But the clip was eight years old*

*(KII-16)*.

A vicious cycle can emerge between social media and traditional media (i.e., newspapers, television news, and radio), where inaccurate reporting by journalists leads to further spread of misinformation and hesitancy and refusal.

*Social media became the triggering cause*, *that doubt* … *whether you should get a vaccine or not*, … *triggered through a false news*, *a false rumor*, *a false thing that is happening in social media*. *And sometimes social media news gets picked up by the print media or the local media gets incited*

*(KII-6)*.

Misinformation that circulates widely is often highly context specific and messages or visuals often seem intentionally targeted at or framed for certain populations.

*People are now receptive to natural medicines*. *It’s increasing due to social media or misinformation or disinformation*

*(KII-10)*.

#### Sociodemographic factors

Vaccine hesitancy presents in diverse communities in India–including across socio-economic status, urban and rural settings, and among different religious communities. Several participants stated that vaccine hesitancy had historically been observed among lower income communities, particularly in the context of polio campaigns.

*It was different earlier in the polio cases [of vaccine hesitancy]*, *most of the resistant families*, *they were poor*. *They were underserved*. *They were not well-educated*

*(KII-20)*.

But during the MR campaign, vaccine hesitancy occurred in high- and low-income communities and was linked to distrust of government services and miscommunication about why children who were previously vaccinated with measles vaccine required a dose of the MR vaccine.

*A trend that we are seeing is* … *the high social and economic and highly educated are against [vaccination] now*

*(KII-10)*.

There were sometimes conflicting perceptions of how vaccine hesitancy differed in urban and rural areas. Hesitancy was described among urban elite and in urban slums, where information spreads rapidly, but also in rural areas, all for different reasons.

*In our urban areas*, *the hesitancy is more among educated people* … *But in rural areas*, *more hesitancy is due to the religious* … *groups*. *Otherwise*, *vaccine acceptance is higher in rural areas compared to urban areas*

*(KII-8)*.

As religion is a powerful force in India, it has the potential to strongly influence attitudes and beliefs towards vaccination for people following many different traditions.

*I see that the section of Christian population that is in the coastal population*, *they don’t believe in vaccination*, *they don’t believe in medicines also*

*(KII-2)*.

Like vaccine hesitancy driven by other factors, hesitancy associated with religion may only pertain to specific vaccines and campaign contexts.

*In routine immunization*, *for example*, *the religion-based resistance is not as high as it was for polio*

*(KII-11)*.

#### Influential leaders, gate-keepers, and anti/pro vaccination lobbies

Influential figures at many levels of society, including high-level political leaders, local community leaders, religious leaders, and celebrities, were recognized as critical determinants of vaccine hesitancy or confidence. Generally, there was recognition that India does not presently have a formal or well-organized “anti-vaccine” lobby; instead, there are powerful voices in communities across India that publicly oppose vaccination, often “opportunists” or “vested interests,” who use media coverage in advance of new introductions or campaigns to promote their position for financial, political, or ideological reasons.

*Certain private people*, *naturopaths*, *were publicly working against this [MR] campaign*. *There’s a strong group in Kerala* … *It’s led by the naturopaths and other systems of medicine*. … *And the purpose is commercial or other interests*

*(KII-8)*.

#### Geographic barriers

Geographic barriers were not a major area of discussion. Participants mentioned that migrant populations were generally accepting of vaccines and health officials faced few issues with vaccine hesitancy in this population, although they can be hard to reach.

*They [migrant communities] are a little hard to reach in the peripheral areas*. *They are working as laborers and often they are migrant*… *So they are often missing their vaccines and not able to complete the vaccination*

*(KII-12)*.

*But we are focusing on them here in Kerala*, *drives [for] special groups [that] focus on migrant people*. … *So there is no problem*. *They are not reluctant*

*(KII-8)*.

*We find there is a very great resistance in the coastal belt*, *among the costal population like fisherman*. *They have Muslims*, *Christians*, *different types of religions are there*, *but as a whole*, *the coastal belt is somewhat resistant to the vaccination*

*(KII-2)*.

However, in Kerala during the MR campaign, widespread refusal occurred among communities where the male head of household worked and live abroad, typically in the Gulf countries, leaving mothers unable to obtain permission or choose vaccination for their child and health workers unable to share information about the benefits of immunization with decision makers.

*One of the main challenges we found*, *in Kerala*, *as you know*, … *one of the members in the family will be abroad*. … *And if we don’t get the consent from the husband*, *the wives may not be able to take their children [for vaccination]*

*(KII-13)*.

#### Historical influences

Participants discussed historical influences in the context of the impact of vaccine hesitancy on previous immunization campaigns. In general, participants thought that vaccine hesitancy had increased recent years, although several noted the lack of data to track trends in beliefs and attitudes about vaccination. Most commented on India’s Pulse Polio Programme, noting some similarities to the MR campaign, including circulation of specific rumors in some communities, and also differences, including occurrence of hesitancy among high-income, well-educated, urban communities in the MR campaign and the new role of social media platforms like WhatsApp in rapidly spreading misinformation.

### C. Vaccine/Vaccination specific issues

#### Design of vaccination program/mode of delivery

According to participants, vaccination campaigns are more susceptible to incidents of large-scale spread of misinformation and hesitancy than the day-to-day UIP operations. Issues are more likely to occur for new vaccines and historically mistrusted vaccines. Campaigns create a media-rich environment where opportunists can use the platform for their own purposes. Certain programmatic aspects of campaigns, such as atypical target population (e.g., MR’s focus on children 9 months to 15 years) or vaccine delivery site locations (e.g., MR’s school-based campaign), can increase risk of miscommunication with the public.

*Any new vaccine campaign is definitely*, *I think*, *toughest of all*, *all three scenarios [for risk of vaccine hesitancy]*. *One is the new introduction*. *There’s a new vaccine coming in*, *people don’t know about it*. *And second is the campaign [mode]*. *[And third is] definitely the target population* … *and that too on very stringent timelines*

*(KII-16)*.

This emphasis should not diminish focus on hesitancy in the UIP; many other drivers of hesitancy cited by participants are common in this setting. For example, participants noted the risk of hesitancy, driven by misreporting of AEFIs in the media and rumors on social media, is a continuous threat facing the UIP.

#### Mode of administration

The mode of vaccine administration did not emerge as a strong theme. Only fear of local reactions related to injections was cited by participants as a reason for vaccine hesitancy.

#### Reliability of vaccine supply

Participants did not see vaccine supply as a strong determinant of vaccine hesitancy; they noted that vaccine availability has increased in recent years through effort of government programs, such as the electronic vaccine intelligence network (EVIN). Some suggested supply issues still occur and can affect specific communities, such as migrant populations or those in urban slums.

*Accessibility is not much a problem in the state of Kerala because we are a small state with high population and all of these decentralized facilities* … *Infrastructure is very good*

*(KII-10)*.

*Sometimes*, *especially [for] those people who are living in the urban slums*, *there’s not a proper structure to deliver the vaccine* … *at a regular interval*

*(KII-20)*.

#### Vaccination schedule, time-investment, and costs

Participants acknowledged that, despite free immunization provided through the UIP, indirect costs could still present a barrier to vaccination for certain disadvantaged populations, such as migrants or day labors.

*[Construction labors may] have to leave their site*, *their a daily rate* … *So there may not be a direct cost*, *but these are indirect costs that are attached to the vaccination that the government provides to them*

*(KII-1)*.

Complicated and changing vaccine schedules can be a source of confusion, concern, and misinformation.

*So*, *these are all questions [that] came up [during the MR campaign]* … “*Why [does] my child need this additional dose*?” *This was the most common question*

*(KII-5)*.

*Since 2000 onwards there were a lot of frequent changes in the [vaccine] schedule*, *so all of these things led to misbelief and mistrust among people*

(*KII-18*)

#### Role of health care professionals

Frontline health workers, specifically accredited social health activists (ASHAs), auxiliary nurse midwives (ANMs) and Anganwadi workers, were identified as a critical source of information and resource for questions about vaccines and vaccination. Pediatricians were recognized as a highly trusted group, although potentially limited by their few numbers, and therefore best utilized through traditional or social media strategies.

*[Frontline workers] entire attitude and their entire interface with the community* … *how they behave*, *how they relate to caregivers*, *matters a lot*. *Those are very big* … *determining factors that influence parents’ decision to not vaccinate or dropout in between vaccinations*

*(KII-21)*.

But participants suggested that these frontline health workers often lack the training to disseminate evidence-based information, respond to vaccine safety concerns, and address misinformation and rumors.

*Health workers*, *especially the grassroot level workers*, *are not able to answer the questions asked by the parents or the public because they are not properly trained*. *So*, *if a person asked that question* “*How does this vaccine prevent a disease*?” *or* … “*What are the side effects of this vaccine*?” *some of the workers are not able to* … *give an answer in a convincing manner*

*(KII-2)*.

Participants also suggested several health systems issues, for example, too few and overburdened health workers, as challenges associated with providing information about vaccination to communities.

*[If frontline health workers] are roped into other activities*, *then this sector is totally neglected*. *And another issue is in urban areas*, *the urban slums*, *[people] are not well cared for by the health workers*

*(KII-20)*.

## Discussion

Our study broadly identified drivers of hesitancy and described complex ways they interact to influence vaccine beliefs, attitudes, and behaviors in India from the perspective of government officials and civil society stakeholders. Understanding these perceptions is critical to inform future vaccine campaigns. Important individual-level influences included low awareness of the benefits of vaccination, safety concerns, especially related to mild AEFIs, and mistrust in government and the quality of health care services. The most critical contextual-level factor was the communications, media, and social media environment, which can serve as a major conduit of misinformation and driver of hesitancy. Vaccine hesitancy was also shown to be shaped by sociodemographic factors, with specific drivers and issues varying widely by income, education, and urban/rural settings, and across religious and cultural groups. Among vaccine/vaccination-level issues, the vaccine program design/delivery and the role of health care professionals emerged as the strongest determinants of vaccine hesitancy and confidence.

In India, routine data are not collected on vaccine attitudes and few studies have explored the determinants of vaccine hesitancy and acceptance [14, 17–19]. Gurnani et al. (2018) reported on a large sample (n = 38,209) of caregivers of undervaccinated children visited for household interviewers through program monitoring in Intensified Mission Indradhanush (IMI) districts from October 2017 to February 2018 [20]. The survey identified mostly individual-level factors as leading reasons for missing vaccination, including lack of awareness (45%), vaccine hesitancy/refusal related to fear of AEFIs (24%), vaccine hesitancy/refusal other than fear of AEFIs (11%), child traveling (8%), operational gaps (4%), and other factors (9%).

A survey of 1,259 random phone numbers across India found that 13% of respondents with children under 5 (n = 288) were vaccine hesitant (n = 36/288) and 2% overall (17% of hesitants) were outright refusers (n = 6/288) [6]. Reasons for hesitancy were largely related to confidence (49%) with fewer due to convenience (18%) or complacency (3%), although almost a third (31%) responded other, don’t know, or no reason. The most common reasons were also related to individual-level factors, such as baby cries/faces problems (n = 6), someone told me/I do not think the vaccine was safe (n = 5), bad experience with previous vaccination (n = 4), and contextual-level factors, including religious reasons/other beliefs/traditional medicine (n = 2).

A mixed-methods study conducted in rural Puducherry after the MR campaign found that 14% of parents of children 9 months to 15 years expressed hesitancy towards this vaccine [12]. Authors concluded that the reasons for hesitancy during the MR campaign were inadequate knowledge about the vaccine, rumors on social media, and inadequate time for campaign planning and implementation.

Our study confirmed, through perspectives of government officials and civil society stakeholders, that for vaccine hesitancy in India, as elsewhere, there is no universal set of factors that determine beliefs and attitudes in every local context [21]. In previous studies, individual-level factors (e.g., vaccine knowledge/awareness or health literacy) are often highlighted as reasons for vaccine hesitancy or low coverage, possibly because they are proximal determinants of a parent’s decision to vaccinate their child [22, 23]. Yet these factors should not be considered in isolation; in fact, parents’ beliefs and attitudes are influenced through their social networks by an array of contextual factors and vaccine/vaccination program factors [24]. Our findings demonstrate the equal importance of contextual and vaccine-specific influences such as the communication and media environment, influential leaders and anti-vaccine voices, socio-cultural differences, historical influences, and the critical role of frontline health workers. Although some research has sought to understand individual-level factors of hesitancy in India, too little is known about contextual and vaccine-specific determinants.

Our discussions with government officials and civil society stakeholders suggested that successful approaches to address specific drivers of hesitancy in India must focus not just on individuals, but on communities, health systems, social media, and external influences. Such approaches should operate across multiple levels of government and society and yet allow for flexibility to adapt strategies, champions, and messages to local contexts, potentially following the cascading design of the country’s vaccine introduction approach, with planning and implementation of different activities at the centre, state, district, and community levels. Focus on vaccine hesitancy in future introduction campaigns is critical; participants shared many lessons learned from the MR campaign (unpublished, Limaye 2021). Participants also emphasized the need for strategies to understand, track, and address vaccine hesitancy in the day-to-day UIP operations, an area they saw as neglected.

This study had limitations. Although we included interviewees from three states in India with diverse experiences related to vaccine hesitancy, their experiences are not representative of all regions and populations in a country as vast and diverse as India, and instead only provide a broad overview of the issues facing the country. As aim of this study was to understand the perspectives of national- and state-level professionals, we lacked interviews with district-level or health facility professionals, local community leaders, or community members, which should serve as the focus of future research. Despite these limitations, because the study participants interviewed, our study provides lessons learned that can help better inform the design and implementation of future vaccine campaigns.

The government officials and civil society stakeholders who implement and support India’s UIP and recent vaccine introduction campaigns are valuable sources of information about the vaccine hesitancy challenges facing communities across the country. Through interviews with these key stakeholders, we identified important drivers of vaccine hesitancy from their perspectives. These data serve as a baseline for further research and programmatic efforts to understand hesitancy and intervene at individual and group, contextual, and vaccine/vaccination-specific levels to address misinformation and promote vaccine confidence in India.

## Supporting information

S1 File (DOCX)
