# Supplementary material for: Understanding determinants of vaccine hesitancy and acceptance in India: A qualitative study of government officials and civil society stakeholders
Source: PLoS One. 2022 Jun 9;17(6):e0269606. doi: 10.1371/journal.pone.0269606 (PMC9182247; doi:10.1371/journal.pone.0269606)
Supplement: S1 File — (DOCX) [file pone.0269606.s001.docx]

**UNDERSTANDING GOVERNMENT AND CIVIL SOCIETY RESPONSES TO VACCINE HESITANCY IN INDIA**

**In-Depth Interview Guide**

**SECTION I: WELCOME**

*Good morning (afternoon). My name is ____. Thank you for coming. I am conducting this interview to understand your understanding of vaccine hesitancy in India. We hope the results of this research will provide useful information for policy and decision makers. I will only ask you about your experiences as a professional. There are no right or wrong answers. Please feel comfortable sharing your full and honest opinions. If it is okay with you, I will be audio-recording our conversation. The purpose of this is so that I can record all of the details but at the same time carry on an attentive conversation with you. All of your comments will remain confidential. In our final report, comments from all interviewees will be listed anonymously.*

[TURN AUDIO RECORDING ON]

**SECTION II: ORAL CONSENT**

[CONDUCT CONSENT PROCESS USING APPROVED ORAL CONSENT SCRIPT]

**SECTION III: INTERVIEW QUESTIONS**

**Part A: Professional Role & Responsibilities**

1. Describe your current and previous experience working in the area of vaccination.

- *With which organizations? In what roles? For how long?*
- *How many years have you been working in the health sector? In vaccination?*

1. With whom do you interact in your work on vaccination?
   - *E.g., health officials, scientists, communications officials, policy makers, etc.?*
   - *Do you interact with community members as part of your role and responsibility? Can you describe this interaction? How frequently?*

**Part B: Vaccine Confidence & Hesitancy in India**

*The WHO Definition of Vaccine Hesitancy:*

*Vaccine hesitancy refers to delay in acceptance or refusal of vaccines despite availability of vaccine services. Vaccine hesitancy is complex and context specific, varying across time, place and vaccines. It is influenced by factors such as complacency, convenience and confidence.*

1. As I’m sure you are aware, the WHO defines vaccine hesitancy as: [Read WHO definition]. Is vaccine hesitancy currently problem in India?
   - *How prevalent is vaccine hesitancy in India?*
   - *Who does vaccine hesitancy affect? (Urban and rural areas? High and low socioeconomic groups? Different cultural or religious groups? Migrant, hard-to-reach, or other disadvantaged groups?)*
2. What are the most common reasons people are hesitant about or refuse vaccines?
   - *Do views differ across communities? How so? By urban/rural, socio-economic status, culture/religion, or disadvantaged groups?*
   - *Do they have different opinions for different vaccines? For campaigns vs. routine immunization?*
   - *Why do people feel this way? What factors drive these opinions?*
3. How about these specific issues: [Read list]? Which are the most common and in which regions or populations do you see them occurring?
   - - *Fear of side-effects (e.g., fever or injection pain)*
     - *Rumors about contaminants or risk of autism*
     - *Rumors about sterilization or plots to control population*
     - *A belief that vaccines are not needed or important*
     - *Head of household lives/works in another location*
     - *Other concerns/rumors/misinformation?*
4. Who do people generally trust most about vaccination and other health issues? For example, if they had concerns, what would be the first source people would go to for information?
   - *Pediatricians? Other doctors? Health workers? News? (TV, paper, radio, etc.?) Internet? Social media (WhatsApp or another?) Other?*
   - *Why do you think people get their information from these sources?*
5. Are other traditional barriers to high immunization coverage still a problem in India? For example, what about: [Read list]?
   - *Product availability (E.g., government stockouts)*
   - *Cost (e.g., hidden costs or private facility fees)*
   - *Travel distance to a facility*
   - *Mother/father/parents being too busy to take the children to a facility*
   - *How mothers and children are treated by health workers*

**Part C: MR Vaccine Introduction Campaign**

1. Can you briefly summarize the planning for the MR campaign, especially related to communications?
   - *What were the specific aims/intended outcomes from the program?*
   - *When did the planning process start? What did it entail?*
   - *Who was involved? Should others have been involved?*

- *In your opinion, do you think the preparation was sufficient? Why or why not?*

1. Can you broadly describe how vaccine hesitancy and refusal occurred in different regions and populations during the MR campaign?
   - *Describe instances where vaccine hesitancy or refusal occurred during the campaign.*
   - *Were challenges related to vaccine hesitancy different by urban/rural, socioeconomic status, culture/religion, or disadvantaged groups?*
   - *How did government and partners respond? What worked and what didn’t?*
   - *What could have been done differently to prevent or better manage these challenges?*
   - *What specifically about the MR campaign led these issues about vaccine hesitancy to arise? Were there vaccine hesitancy-related concerns prior to the MR campaign? What underlying factors contributed to these issues?*
2. What was the role of social media in the MR campaign? How did platforms such as WhatsApp and Facebook affect the campaign?

- *Please describe the types of messages that circulated. Where did they circulate?*
- *How did government and partners respond? How did it go?*
- *What could have been done to prevent circulation of these messages or improve the partner response once they began circulating?*

1. Did the MR campaign have a lasting impact on people’s attitudes/behaviors towards vaccination?
2. Have government or partners changed their thinking or practice related to vaccination after the MR campaign?
   - *I.e., what were the “lessons learned”? Have they been implemented?*
   - *What more could be done?*

**Part D: Recommendations**

1. What is being done nationally or in states by government or partners to counter vaccine hesitancy and refusal?
   - *How did these programs come about?*
   - *Are they effective?*
2. Could you suggest how we might improve public confidence in vaccines?
   - *Think broadly about possible measures. Media campaigns? Community meetings? Social media messaging? Incentives? Messaging at schools? Improved treatment by health workers? Local leaders speaking out? Celebrity champions?*
3. Is there any other information that you would like to share?
4. Could you recommend any other professionals who might participate in this discussion?

**SECTION IV: THANK YOU**

Thank you very much for completing this interview. Your time is very much appreciated, and your comments have been very helpful.

[TURN AUDIO-RECORDING OFF]
